# Exploring a core psychopathology in disordered eating: the feelings of fat scale

**DOI:** 10.1186/s40337-021-00401-z

**Published:** 2021-06-02

**Authors:** Yichelle Y. Zhang, Bruce D. Burns, Stephen Touyz

**Affiliations:** School of Psychology, The University of Sydney, Sydney, NSW 2006 Australia

**Keywords:** Eating disorders, Feelings of fat, EDE-Q, Scale development

## Abstract

**Background:**

Feelings of fat are common for people with eating disorders, but ways of measuring its intensity are needed. Therefore, our goal was to develop a self-report feelings of fat scale that asked participants to indicate how intensely they currently felt statements such as “I feel fat”. With such a scale we can determine how strongly feelings of fat relate to evidence of disordered eating.

**Methods:**

We conducted three studies of eating disorders with undergraduate women taking introductory psychology classes. The combined sample was 472 participants. A previous eating disorder diagnosis was self-reported by 33 participants and a current diagnosis by 11. All participants completed the EDE-Q and the seven (Study 1) or nine item (Studies 2 and 3) “State Feelings of Fat” (SFF) scales we developed. Each item asked them to rate the intensity with which they felt statements such “I feel fat” on a seven-point scale from “not at all” to “the most I have ever felt”.

**Results:**

Both the seven and nine item SFF scales were highly coherent (Cronbach’s α were .94, .95 and .94), but factor analysis supported the seven-item version. We found high correlations between SFF and EDE-Q scores (Study 1: .816; Study 2: .808; Study 3: .841). SFF scores distinguished participants self-reporting no eating disorder diagnosis from those with a former diagnosis, *t* (361) = 2.33, *p* = .021, who in turn were distinguished from those with a current diagnosis, *t* (42) = 2.09, *p* = .043. Due to the high coherence of the scale, the single item “I feel fat” captured most of the variance in EDE-Q scores (*r* [472] = .793).

**Conclusions:**

We have constructed an eating disorders relevant feelings of fat scale. Given that the EDE-Q is considered a valid questionnaire for measuring severity of eating disorders, our findings suggests that feelings of fat are core to the psychopathology of eating disorders. To the extent that EDE-Q scores are stable it also suggests that feelings of fat are surprisingly stable. Furthermore, the single item “I feel fat” alone may capture most of what the EDE-Q measures.

## Plain English summary

Many people with eating disorders report feelings regarding fat. In this paper we developed a way to measure how intensely someone has such feelings and tested how strongly this was related to potentially having an eating disorder (as measured by the EDE-Q scale). Our questionnaire had seven items which asked how intensely a person felt statements such as “I feel fat”. In three studies with a combined sample of 472 university women we found our scale to be reliable and that the association between our *feelings of fat* scale and EDE-Q scores was a .818 correlation. Thus, asking about the intensity of feelings of fat appears to be strongly associated with how likely someone is to have a potential eating disorder. This suggests that feelings of fat are an important element of eating disorders.

## Introduction

“I’m feeling (so) fat” is a phrase many women, and increasingly men, voice on a regular basis. Striegel-Moore, McAvay, and Rodin [1] presented evidence that feeling fat is a common phenomenon, El Ansari, Clausen, Mabhala and Stock [2] found that over 60% of women (and almost 40% of men) in a sample of English and Danish students reported feeling “too fat” and Cooper, Deepak, Grocutt, & Bailey [3] found that feeling fat was present in those with and without a diagnosed eating disorder. In clinical settings, practitioners have observed that for people with an eating disorder feelings of fat are experienced with greater intensity and frequency, and can fluctuate significantly in very short periods of time (Cooper, et al. [3]; Fairburn & Beglin [4]; Murphy, Straebler, Cooper, & Fairburn [5]). Linardon et al. [6] reported high correlations between the single “I feel fat” item from the EDE-Q and that measure’s restraint (.61) and eating concerns subscales (.58) for a sample of participants diagnosed with anorexia nervosa or bulimia nervosa. The “I feel fat” item accounted for unique variance even after taking into account diagnosis, depressive symptoms and scores on an over-evaluation scale. Linardon et al. point out that “feeling fat” has received scant research attention even though it is featured prominently in theoretical models of eating disorders (Fairburn, Cooper, & Safran [7]). They suggest that more research is needed and that scales for measuring such feelings need to be developed. In this paper we report the development of a feelings of fat scale and use undergraduate samples to test it across the full range of self-reported disordered eating as measured by the EDE-Q.

For many people with an eating disorder the feelings they have about fat are complex (Major, Viljeon, & Nel [8]). Early studies suggested that feeling fat can be largely driven by psychological factors (e.g., depressed affect, Tiggemann [9]), and that it is different to being objectively overweight (Striegel-Moore et al. [1]). It has been posited that feelings of fat may be a mislabelling of other emotions as a result of body displacement, whereby individuals transform negative affect into feelings about the body (e.g., Bruch [10]; Elderedge, Wilson & Whaley [11]; Taylor & Cooper [12]; Coelho, Carter, McFarlane, & Polivy [13]; McFarlane, Urbszat & Olmsted [14]; MacDonald [15]). Based on clinical observations, Murphy, at al. [5] speculate that negative emotions (such as depression, boredom, distress, anxiety, etc) or physical sensations (such as feeling full or bloated) may drive feelings of fat, rather than physical changes in fat itself.

Major et al. [8] used semi-structured interviews of seven women with an eating disorder diagnosis and who reported experiencing feeling fat in order to try to understand these feelings. All participants spoke about their awareness of both internal and external physical sensations and experiences, and analysis identified four superordinate themes: 1. Negative sense of self (e.g., “[Feeling fat] means … I’m incompetent, I’m lazy and I don’t have self-control or self-discipline, that I’m physically unattractive, that I’m a bad person”); 2. Feeling out of control (e.g., “I’m not very good at being aware of my emotions … sometimes complicated difficult emotions get condensed in my mind into ‘I feel fat’”); 3. Coping with feeling fat (e.g., “[When working] I didn’t feel fat crawling up me, I didn’t feel any of that, as long as the next day I was able to be up, at work early and perform”); 4. Making sense of feeling fat is complex (e.g., “I think I sort of attach it to a lot of different things [long pause] so it’s difficult to pin down exactly what it is”). Six of the seven participants explained that it was hard to put into words their experience of feeling fat, and it is because of this complexity that we favour the term “feelings of fat” rather than “feeling fat” or “feelings of fatness.” The latter two terms could be interpreted as just referring to feeling large, but “feelings of fat” captures the idea that people with an eating disorder can have complex emotions around fat. Feelings of fat does not impose a particular view as to what those feelings are, so this is the term we will use in this paper.

### Measuring feelings of fat

A major difficulty for evaluating the evidence regarding the role of feelings of fat in eating disorders is the variation in the ways feelings of fat have been measured, which highlights the lack of a direct empirical measure for this construct. Sometimes indirect measures such as body dissatisfaction scales (e.g., Tiggemann [9]; McFarlane et al. [14]), thought-shape fusion measures (e.g., Lobera, Santed, Rios, Prieto, Fernandez & Casals [16]) and implicit measures such as the Implicit Association Task (e.g., McDonald [15]) have been utilised in the measurement of feelings of fat. Whilst Striegel-Moore et al. [1] report having developed a seven-item fat questionnaire, the full scale was not described. Consequently, the main aim of the current research was to develop and validate a coherent scale that captures an individual’s current feelings of fat, the State Feelings of Fat (SFF) scale. Following the work linking feelings of fat with emotion we assumed that such feelings can be measured like other emotions, that is, through self-report.

### Approach

For this study we used samples of women studying at university. Linardon et al. [6] found evidence that degree of feeling fat could be differential for women diagnosed with an eating disorder, but an effective measure of feelings of fat should be related to degrees of disordered eating across the full spectrum of disordered eating. Therefore, an undergraduate university sample was better for scale development, although this might initially limit generalizability to populations beyond this group. We restricted the study to women because feelings of fat may play a different role for women than for men and so we focused our resources on women only. Whilst the question of what role feelings of fat plays for men is an interesting one, it is an issue we left for future studies.

Given the absence of existing validated feelings of fat scales against which to validate our scale, we needed to decide on a strategy for validating the SFF scale. One way to validate it was to test the hypothesis that the scale should correlate with measures of eating disorders. As discussed above, previous research has suggested that feelings of fat are associated with eating disorders. To this end, we utilised the EDE-Q (Fairburn & Beglin [17]) which is an easy to administer questionnaire for measuring disordered eating derived from the EDE (Cooper, Cooper, & Fairburn [18]), and is widely used for assessing eating disorders (Berg, Peterson, Frazier & Crow [19]) although it is not without criticisms. Berg, et al.’s systematic review of the EDE-Q found that there is good evidence of its reliability and validity. Although it cannot be used to make a diagnosis itself, it has been found to strongly discriminate between people with or without an eating disorder diagnosis (Mond, Hay, Rodgers, Owen, and Beumont [20]; Mond, Myers, Crosby, Hay, Rodgers, Morgan, Lacey & Mitchell [21]; Aardoom, Dingemans, Slof Op’t Landt & Van Furth [22]). Therefore, we considered the EDE-Q a useful measure against which to validate the SFF scale.

## Study 1

Our first version of the SFF consisted of seven statements. These were constructed by taking into account bodily sensations identified in Cooper et al. [3] associated with feeling fat (such as “clothes feeling tighter”, “feeling heavy”, and “thighs are thick and heavy”) which directly led to three items, from informal discussions with individuals who have eating disorders and from statements observed in publicly accessible social media platforms. These observations predated this study and were not part of a formal study, so we cannot report any specific findings. However, based on these informal observations, we identified broad items such as “I feel fat”, and more specifically physical statements such as “I feel that my body is distended”. For each statement participants indicated how intensely they felt each statement in the present moment. All scale items can be seen in Table 1. Our scale has some overlap with Striegel-Moore et al’s [1] scale because the only two items they describe, “Do you feel fat now” and “How do you feel about your thighs”, are similar to two of our items.

**Table 1:** Means, standard deviations and correlation coefficient for SFF and EDE-Q scores as well as the seven individual SFF items used in Study 1. All correlations were statistically significant at *p* < .001 (*N* = 213)

	EDE-Q	SFF score	Item 1	Item 2	Item 3	Item 4	Item 5	Item 6	Item 7
SFF score	.816								
1. I feel that my clothes are tight	.553	.715							
2. I feel that my stomach is distended	.673	.814	.586						
3. I feel that my face is round and chubby	.634	.801	.511	.629					
4. I feel my thighs are thick and heavy	.687	.857	.563	.619	.607				
5. I feel my entire body is wide	.737	.913	.547	.670	.682	.754			
6. I feel big	.777	.933	.584	.714	.667	.769	.886		
7. I feel fat	.791	.920	.579	.673	.668	.762	.858	.906	
*Mean*	2.26	3.18	2.59	3.27	3.28	3.79	3.11	3.05	3.19
*SD*	1.32	1.50	1.42	1.60	1.81	1.86	1.86	1.86	1.88

Note. This table reports Pearson product-moment correlations, as are all the correlations reported in this paper. Spearman correlations would have tested the hypothesis of monotonicity rather than linearity (see de Winter, Gosling, & Potter [23], for a discussion). Given that we are using EDE-Q to try to validate SFF, linearity is the appropriate hypothesis to test. In practice, Spearman correlations for this data differ very little from Pearson correlations

As well as the SFF participants completed the EDE-Q. As well as exploring the statistical properties of the SFF we tested how strongly it correlated with EDE-Q scores.

### Method

#### Participants

139 women taking introductory psychology at the University of Sydney and 76 women taking introductory psychology at the University of NSW completed the experiment for partial course credit. Their average age was 18.9 years old (*SD* = 2.4) and 95% were 22 years old or younger. Participants had a mean BMI of 21.9 (*SD* = 3.77; Range 16.7 to 34.9). This and subsequent research involving University of Sydney students was approval by the University of Sydney Human Research Ethics Committee and research involving University of NSW students was approved by the UNSW Human Research Ethics Committee.

#### Materials

We gave participants the 36-item EDE-Q (Fairburn and Beglin [17]) and our State Feelings of Fat (SFF) scale. For the SFF scale participants were presented with a page on which was written “This scale measures how you feel right now about your body. On a scale of 1 to 7, with 1 being not at all and 7 being the most you have ever felt this way, please indicate the number under each question that most corresponds with how you are feeling right now, that is, in this present moment.” They then read the seven statements listed in Table 1 and for each the seven possible responses were numbered and labelled as follows: 1 ‘not at all’, 2 ‘a little’, 3 ‘slightly’, 4 ‘moderately’, 5 ‘strongly’, 6 ‘extremely’, 7 ‘the most I have ever felt’. We labelled the extreme end of the scale as ‘the most I have ever felt before’ because we wanted this scale to be able to capture the most intense feelings of fat that a participant can feel. We expected this response category to be rarely used. Note that whereas it is possible that someone who never felt fat at all could label their current feeling as ‘the most I have ever felt before’ we considered this interpretation of the response scale to be unlikely given the location of this response at the extreme right of the scale and the presence of responses that would less ambiguously capture such feelings.

#### Procedure

Participants chose this study from an online menu of possible studies. In this menu this study was referred to as “How Do I Feel About My Body?” and the amount of course credit they would receive for completing the study was stated. The sign-up system only allowed women to see the study. Gender identification was based on self-identification in a pre-screen survey given to all members of the participant pool the first time they accessed the system. As part of the pre-screen survey, the University of Sydney students also completed the five-item SCOFF screen for eating disorders (Morgan, Reid, & Lacey [24]). Using these responses, we were able to bias our sample by manipulating the availability of time slots such that half our participants met the SCOFF criterion for having a potential eating disorder (i.e., responding yes to two or more SCOFF items) and half did not meet the criterion. A sample drawn more evenly across the spectrum of disordered eating will have better statistical properties and thus be better for scale development.

Upon arriving at the lab participants read information about the experiment and, if they consented to participate, they completed the 36-item EDE-Q (Fairburn & Beglin [17]) and our SFF scale. The scales were given at the beginning of an experiment that tested other hypotheses than those that are the focus of this paper. We will not report the procedure that followed the completion of the scales, except that at the end of the experiment participants were weighed with a scale that did not show the participants their weight and they self-reported their height. A tape measure was available if they were not sure of their height.

After running the first 100 participants from the University of Sydney we added a question to the end of the procedure which asked whether the participant had a current or previous eating disorder diagnosis, or had never received a diagnosis of an eating disorder. Participants were not asked to specify the nature of any diagnosis and we could not verify their responses. Only a small number reported a current or previous diagnosis so we will not analyse them in Study 1. However, across all three studies reported here enough participants reported a diagnosis that we could analyse them, which we do below in the section in which we combined the samples.

### Results and discussion

Two participants did not complete all items, so they were excluded from the analysis. For each participant we calculated an EDE-Q global score in the standard way, which was to find their mean response to items 1–15 and 29–36 (this excludes items asking about the frequency of compensatory behaviours). Each item was scored on a scale of 0–6, so global EDE-Q scores were also in the range 0–6 (the range for this sample was 0.00 to 5.22). We used the global score although Linardon et al. [6] used two subscales of the EDE-Q because attempts to find statistically significant support for the subscales have failed (Aardoom et al., [22]; Mantilla, Birgegård & Clinton [25]). Although we collected responses to the compensatory behaviours questions on the EDE-Q we did not analyse this data because Linardon et al. found only weak relationships between them and their other measures. SFF scale scores were calculated by averaging a participant’s responses (scored 1–7) over the seven items, and the full range of 1.00 to 7.00 was observed in this sample. Table 1 presents the means of each of the EDE-Q and SFF scores, the means for each SFF item, and the correlations between all these measures.

Two notable finding about the SFF scale are clear from Table 1. First, SFF scores are highly correlated with EDE-Q global scores such that SFF scores capturing over 66% of the variance in EDE-Q scores. Second, as can be seen from Table 1, all SFF items were highly correlated; so, the SFF scale was highly coherent (Cronbach’s α = .94) indicating good reliability. Despite seemingly large variations in their wording, it was hard not to conclude that the SFF items are each largely measuring the same construct. So we appear to have evidence that our SFF scale was reliable and that it was valid in that it correlated very highly with EDE-Q scores.

The SFF scores also correlated with BMI, *r* (213) = 0.471, *p* < .001, which also correlated with EDE-Q global scores, *r* (213) = 0.360, *p* < .001. However, accounting for the common variance of these two variables with BMI, the partial correlation of SFF with EDE-Q was only slightly reduced, *r* (213*)* = .786, *p* < .001.

## Study 2

Study 2 was an attempt to replicate the surprisingly high correlation between EDE-Q and SFF scores. However, we added items to the SFF scale.

In deciding how to label the responses to items on the SFF scale we chose to label the high end of the response scale “the most I have ever felt” with the expectation that such a response would rarely be used and thus the SFF would be sensitive to the full range of feelings of fat intensity. We expected it to be rare for a participant walking into the lab to describe any feeling as “the most I have ever felt”, yet this extreme response was used quite regularly by participants in Study 1. For example, the “I feel fat” item drew this response from 11.5% of participants. Therefore, we were concerned that the SFF may effectively have a ceiling that would prevent it from being sensitive to the full range of feelings of fat. To address this possibility we added two additional items that would seem to be able to capture extremely heightened feelings of fat. In particular, the items tried to tap into the visceral, internal way that fat could be felt. We hoped that such items would add to the scale’s discrimination.

### Method

#### Participants

149 women taking introductory psychology at the University of Sydney participated for partial course credit. They were recruited in the same way as in Study 1 and again pre-screening responses to the SCOFF were used to bias the sample towards participants with evidence of potentially disordered eating. Their average age was 19.0 years old (SD = 2.26) and 95.9% were 22 years old or younger. Their mean BMI was 21.4 (*SD* = 2.97; Range 14.4 to 32.5). So the sample was is similar to that used in Study 1.

#### Materials and procedure

The materials and procedure were identical to Study 1, except that the SFF scale was augmented with two additional items: “8. I feel like the cells in my body are turning into fat” and “9. I feel like blobs of fat are attaching themselves to my body”. These items were again derived from informal discussion with people with eating disorders and social media posts.

### Results and discussion

For each participant global EDE-Q and SFF scores were calculated as in Study 1, except that SFF scores were now averaged over nine items. The Table 2 descriptive statistics and correlations for these scores for the items making up the SFF were very similar to Study 1, and EDE-Q and SFF scores were similarly highly correlation. If SFF was calculated based on just the seven items used in Study 1 the correlation was virtually identical, *r* (149) = .816, *p* < .001. Therefore, we replicated the high correlation found in Study 1.

All SFF items, including the two new ones, were again highly correlated so they formed a highly coherent scale (Cronbach’s α = .95). Adding the two items to SFF did result in no participants having the maximum SFF score with its range being 1.00 to 6.86.

## Study 3

The two previous studies found that the SFF was a highly coherent scale to such an extent that a single item might capture most of the predictiveness of EDE-Q scores. The simple item “I feel fat” correlated with EDE-Q scores *r* (213) = .791 in Study 1 and *r* (149) = .786 in Study 2. However, this might be because it appeared on a page with all the other feelings of fat scale items. So, in Study 3 we tested whether isolating this one item and giving it either before or after the other items would affect its statistical characteristics. In addition, we sought to again replicate the high correlation of SFF with EDE-Q scores.

### Method

#### Participants

110 women taking introductory psychology at the University of Sydney participated for partial course credit. Recruitment was the same as in Studies 1 and 2. Their average age was 19.7 years old (*SD* = 3.08) and 90.1% were 22 years old or younger. Their mean BMI was 21.5 (*SD* = 3.00; Range 15.8 to 31.0).

#### Materials and procedure

The procedure was the same as Study 2 and used the nine item SFF scale. However, the “I feel fat” item was presented alone on a separate page to the other eight SFF items. Participants were randomly assigned by the computer presenting the materials to an *Order* factor with two levels, either *Before* or *After* conditions which determined whether the single “I feel fat” item was presented immediately before or after the other eight SFF items.

### Results and discussion

For each participant EDE-Q and SFF scores were calculated as in Study 2. Table 2 shows that EDE-Q and SFF scores were again highly correlated, *r* (110) = .841, *p* < .05. All SFF items were again highly correlated so they formed a highly coherent scale (Cronbach’s α = .94).

**Table 2:** Means and standard deviations for SFF, EDE-Q scores and individual SFF items for Studies 2 (*N* = 149) and 3 (*N* = 110). Study 2 correlations are in the lower half of the table and Study 3 correlations in the upper half. All correlations statistically significant at *p* < .001

	EDE-Q	SFF	Item 1	Item 2	Item 3	Item 4	Item 5	Item 6	Item 7	Item 8	Item 9
EDE-Q	–	.841	.633	.679	.643	.675	.775	.830	.824	.622	.605
SFF score	.808	–	.764	.812	.811	.822	.921	.932	.908	.758	.738
1. I feel that my clothes are tight	.525	.678	–	.682	.605	.567	.659	.674	.658	.448	.450
2. I feel that my stomach is distended	.693	775	.628	–	.690	.592	.742	.751	.741	.464	.411
3. I feel that my face is round and chubby	.614	.761	.416	.574	–	.693	.692	.716	.687	.463	.478
4. I feel my thighs are thick and heavy	.693	.875	.535	.656	.640	–	.755	.753	.733	.515	.500
5. I feel my entire body is wide	.742	.925	.556	.656	.701	.806	–	.915	.873	.623	.611
6. I feel big	.763	.915	.561	.665	.637	.775	.925	–	.869	.649	.627
7. I feel fat	.786	.927	.552	.687	.672	.817	.877	.908	–	.637	.586
8. I feel like the cells in my body are turning into fat	.589	.793	.478	.499	.494	.635	.654	.649	.670	–	.875
9. I feel like blobs of fat are attaching themselves to my body	.590	.698	.473	.489	.527	.662	.679	.658	.689	.825	–
Study 2 Mean (SD)	2.39 (1.4)	3.09 (1.5)	2.81 (1.4)	3.38 (1.6)	3.43 (1.8)	3.89 (1.9)	3.19 (2.0)	3.10 (2.0)	3.19 (1.9)	2.56 (1.8)	2.29 (1.8)
Study 3 Mean (SD)	2.49 (1.5)	3.07 (1.5)	2.94 (1.7)	3.44 (1.7)	3.56 (2.0)	3.76 (1.9)	3.15 (1.9)	3.10 (1.9)	3.39 (1.7)	2.37 (1.9)	1.96 (1.6)

We examined the effect of order on the “I feel fat” item. Mean “I feel fat” ratings were slightly lower for *Before* condition participants (*M* = 3.21, *SD* = 1.60, *N* = 56) than for *After* condition participants (*M* = 3.57, *SD* = 1.76, *N* = 54), but the difference was not statistically significant, *t* (109) = 1.123, *p* = .264. The correlation between EDE-Q scores and “I feel fat” was similar for the *Before* (*r* [56] = .803) and *After* (*r* [54] = .847) conditions, *z* = 0.706, *p* = .24. These correlations were slightly higher than those between EDE-Q scores and this item in Study 1 and Study 2, so there was no evidence that separating the “I feel fat” item from the rest of the SFF scale lowered its association with EDE-Q scores.

### Analysis across samples

The three studies established the validity of the SFF scale as a predictor of EDE-Q scores across the full range of EDE-Q scores. All studies used the same procedure (except for the addition of two items), recruited from equivalent pools of participants, and found virtually identical results. So, to address further questions we combined the samples for analysis.

For the combined sample the correlation between EDE-Q and SFF was high for the nine-item SFF, *r* (258) = .820, and for the seven-item version, *r* (472) = .818.

### Diagnoses and SFF

Our samples were all recruited from first year psychology classes and fortunately there were only a small number of participants with diagnosed eating disorders in each sample. However, by combining across studies we had an analysable sample of participants who had received an eating disorder diagnosis. Out of 376 participants, 11 (2.9%) reported a current diagnosis of an eating disorder, 33 (8.8%) reported a former diagnosis, and 332 (88.3%) reported no diagnosis. We could not verify the accuracy of these self-reports and given how high were the EDE-Q scores of some participants who reported no diagnosis it was likely that some participants chose not to report a diagnosis or were diagnosable. However, we believe there was enough validity to participants’ responses to allow us to contrast these groups. All participants asked the diagnosis question completed at least the first seven items of the SFF, so we will use the seven-item SFF scores for this analysis.

Table 3 shows that there was little difference between diagnosis groups in terms of age or BMI. Unsurprisingly the never diagnosed group had a lower mean EDE-Q score than the former diagnosis group, *t* (361) = 3.89, *p* < .001. In turn, the former diagnosis group had lower EDE-Q scores than the current diagnosis group, *t* (42) = 2.84, *p* < .001. The same pattern was present for mean SFF scores with the never diagnosed group lower than the former diagnosis group, *t* (361) = 2.33, *p* = .021, who in turn had lower SFF scores than the current diagnosis group, *t* (42) = 2.09, *p* = .043.

**Table 3:** Means for age, BMI, EDE-Q global scores and SFF scores (standard deviations in parentheses), plus the SFF correlation with EDE-Q global scores, for participants divided by their response to the diagnosis question (all correlations statistically significant, *p* < .05)

	Never diagnosed (*N* = 328)	Former diagnosis (*N* = 33)	Current diagnosis (*N* = 11)
Age	19.3 (2.7)	19.15 (1.62)	19.73 (2.97)
BMI	21.78 (3.10)	22.05 (2.95)	22.03 (4.42)
EDE-Q global	2.21 (1.30)	3.14 (1.48)	4.46 (0.70)
SFF score	3.16 (1.49)	3.80 (1.79)	5.01 (1.19)
Correlation of SFF with EDE-Q	.800	.869	.632

As can be seen from Table 3, EDE-Q and SFF were highly correlated even within the former and currently diagnosed groups. Thus, even for participants reporting a clinical diagnosis of an eating disorder their SFF scores are highly predictive of their degree of disordered eating, similar to what Linardon et al. [6] found for a group with anorexia nervosa and bulimia nervosa diagnoses. The lower correlation for the currently diagnosis group compared to the other two could largely be a product of the restricted range of SFF scores for them. The minimum SFF score for participants with a current diagnosis was 2.86 but nine out of eleven such participants had scores above 4.7 (where 5 represents “strongly”).

### Feeling of fat across demographic categories

A limitation of our studies was that all participants were undergraduate women in Sydney which raises questions about the generalizability of our results. We acknowledge this as a limitation, however there was some heterogeneity in our samples, in particular, the ethnic background of our participants was somewhat varied.

The majority of our sample were native English speakers but there was a large number of participants with non-English speaking backgrounds in our study. The largest non-native English speaker group in our studies were those reporting that Chinese was their first language, although we don’t know which of these were Australian residents from immigrant families and which were non-residents studying on student visas. There have been a large number of Chinese immigrate to Australia over the last 30 years and a large number of students from China study at the University of Sydney. The 99 Chinese native speakers reported a similar correlation between SFF (based on the first seven items) and EDE-Q (*r* [99] = .800) as the full sample (*r* [472] = .818). Therefore, there is some evidence that our findings generalize to Chinese speaking students.

### Which items do we need for the SFF?

The high coherence of the SFF scale and the items’ high inter-correlations suggests that perhaps not all nine items were necessary. To see if we could trim the number of items, we conducted a factor analysis to see if items may load on different factors.

Using the data for the nine-item version of the SFF from Studies 2 and 3, a principal axis factor analysis was conducted using IBM SPSS Version 26 with oblique rotation (oblimin). The Kaiser-Meyer-Olkin measure verified the sampling adequacy, KMO = .916, and KMO values for individual items of at least .844. The first factor accounted for 69.1% of total variance and is the only one with an eigenvalue above 1.0, but the scree plot supported retaining two factors. Table 4 shows the loadings for each factor after rotation.

**Table 4:** Eigenvalues, percent variance explained for the two extracted factors, together with factor loading for SFF items 1–9 after rotation

	Factor 1	Factor 2
Item 1	.688	−.010
Item 2	.890	−.136
Item 3	.769	−.021
Item 4	.773	.082
Item 5	.863	.096
Item 6	.861	.096
Item 7	.843	.107
Item 8	.060	.857
Item 9	−.001	.935
Eigenvalues	6.218	0.873
% of variance	69.1%	9.7%

This analysis found that there was one dominant factor that accounted for most of the variance in the item responses, but that the two items we added in Study 2 may load on a separate factor. These two items have lower item correlations with EDE-Q scores in Table 2 than the other seven, suggesting that they may be less effective items. This suggests we should drop these items from the SFF.

### Could a single item be used?

The factor analysis of the SFF in Table 4 found that a single factor accounting for most of the variance in EDE-Q scores, and that all seven items loaded very highly on this factor. This raises the question of whether we could measure feelings of fat with just the simplest item “I feel fat”, which correlates .793 with EDE-Q scores in the combined sample.

The high correlation of “I feel fat” with EDE-Q scores suggest that they map strongly to each other. The strong mapping of responses to this item to different levels of EDE-Q scores is illustrated in Table 5 which shows the means and standards of EDE-Q scores for participants giving each level of response to the “I feel fat” item. Each increased level of response was associated with a clear increase in mean EDE-Q scores.

**Table 5:** Mean EDE-Q global scores for each level of response to “I feel fat” (total sample 472)

Response:	not at all	a little	slightly	moderately	strongly	extremely	the most I have ever felt
Mean	0.98	1.72	2.19	2.74	3.57	3.97	4.36
*SD*	0.77	0.82	0.90	0.94	0.86	0.73	0.79
*N*	117	85	56	86	60	46	22

This analysis raises the intriguing possibility that this single item alone could be used to screen participants likely to have high EDE-Q scores and thus likely to have a diagnosable eating disorder. Aardoom et al. [22] and Mond et al. [21] found evidence that an EDE-Q global score greater than 2.8 was the best discriminator between people with and without an eating disorder. Based on this criterion, Table 5 suggests that a response to “I feel fat” of at least “strongly” could be a potential screen for an eating disorder.

### “I feel fat” verse “Have you felt fat”

Feelings of fat was assessed by Linardon et al. [6] using the item from the EDE-Q that directly addresses it: “Have you felt fat?” Like all EDE-Q items, participants answered this in terms of number of days this occurred in the last 28 days (from “no days” to “every day”). In contrast, SFF asked about current intensity, so they ask about very different time scales. However, the single EDE-Q item correlated very highly with the seven-item SFF, *r* (472) = .792, *p* < .001. The EDE-Q item was also correlated highly with the single SFF item “I feel fat” despite the difference in time scale, *r* (472) = .815, *p* < .001, suggesting that intensity and frequency of feeling fat were highly correlated. Our examination of this EDE-Q item was theoretically driven, not a quest to find the highest correlated single items, but in our sample this item had the highest correlation of any single EDE-Q item with EDE-Q global scores, *r* (472) = .838, *p* < .001.

## Discussion

The initial goal of this study was to develop a scale for measuring an individual’s current state of feelings of fat. We have succeeded in creating a coherent seven item scale for measuring this. However the most surprising finding to emerge from our studies was just how highly correlated feelings of fat were with EDE-Q scores, which are considered a valid measure of disordered eating. To give some context to the combined sample .818 correlation we found for the SFF, it is higher than the correlation between the most common intelligence tests, the WAIS, Stanford-Binet and Raven’s Progressive Matrices (Guertin, Ladd, Frank, Rabin, & Hiester [26]). Therefore the correlation was high enough to suggests that our SFF scale and the EDE-Q are measuring the same underlying construct. The EDE-Q also asks directly about feeling fat, but this item is just one item amongst items that variously address restraint, eating concerns, weight concerns and shape concerns. Yet participants’ responses to this one item captured most of the variance in the full EDE-Q scale. To the extent that the EDE-Q is a valid indicator that someone may have an eating disorder, our results together with Linardon et al’s [6] results imply that feelings of fat are more central to eating disorders than has been recognized. It may not be surprising that participants with high EDE-Q scores or having a diagnosis of an eating disorder had high feelings of fat scores. However, our results also showed that despite the evidence that feeling fat is wide-spread in our culture (Striegel-Moore, et al. [1]), women not presenting evidence of an eating disorder (as indicated by low EDE-Q scores) were clearly distinguished by much less intense feelings of fat.

Another implication of the high correlation between feelings of fat and EDE-Q scores is that it suggests that degree of feelings of fat are more stable than suggested by Fairburn [4]. The EDE-Q is designed to measure a stable characteristic of a person (evidence of the presence of an eating disorder) yet SFF items asking about feelings right now are correlated highly with this presumably stable measure. However, even if feelings of fat are stable, this does not mean that they are always front of mind. These feelings may always be there ready to be made explicit by events or a questionnaire.

If feelings of fat are stable and central to disordered eating behaviour then the question of exactly what feelings of fat are and what role they play in eating disorders rises in importance. In the EDE-Q the item “I feel fat” fell on the shape concern subscale identified by Fairburn [4]. Our SFF scale has items asking about the participants feelings about their stomach and about their thighs, and similarly Garner, Olmstead and Polivy’s [27] Eating Disorder Inventory has question about these body parts that fall on their body dissatisfaction subscale. However, Linardon et al. [6] suggest that evidence is growing that there are distinct attitudinal components of body image that may function differently with regard to eating disorders. Exactly how feelings of fat fit within these distinctions is an issue requiring more exploration, as is the complexity that Major et al’s [8] qualitative study points to for such feelings.

Our theoretical motivation for focusing on feelings of fat was that it could be a substitute for emotions. A more general framework for the idea that escape from emotions could play a key role in eating disorders was explored by Heatherton and Baumeister [28]. They proposed that binge eating may be an escape from a self-awareness that has brung the sufferer feelings of threat, anxiety or depression. They also discuss perceptions of fatness but as a cause rather than a substitute for such anxieties. Our results and other recent finding regarding feelings of fat suggest that it is time to explore more carefully the role they may play in disordered eating. By provide a scale to measure feelings of fat, this paper can help that exploration.

### Limitations

One limitation of the SFF was that SFF items are self-report, but this is also true for the EDE-Q which has been extensively validated (Berg et al. [19]). Ideally, we would like to develop measures of feelings of fat that would be less subjective. A more critical limitation of our findings is that all our participants were female university students in Sydney, which raises the question of how generalizable our results are. However, there was some diversity within our sample which regards to ethnicity, and our results stood up across this variable. There is a need to investigate the SFF scale in a non-university sample.

Although the high Cohen’s kappa results indicate that our scale is reliable, we did not examine test-retest reliability. This was because we intended the scale to be a state measure which we expected to fluctuate over time. The high correlation between SFF and EDE-Q scores suggest both that SFF is more stable than expected, and that SFF would have reasonable test-retest reliability. However this needs to be tested. Whether we failed to construct a state measure, or the SFF is a state measure of a state that is usually stable, will require more research.

Another possible limitation is that we only used the EDE-Q as our measure of disordered eating, whereas there are a number of other measures we could have included. The EDE-Q is often used as the sole measure of eating disorder symptoms in research into eating disorders (Luce, Crowther and Pole [29]; Mond, Hay, Rodgers and Owen [30]). However whether the SFF would correlate as highly with other measures of eating disorders is a question needing further investigation.

### Clinical significance

Our results have potentially important clinical implications. Our analysis of participants reporting a current or former diagnosis of an eating disorder found that not only did these groups have higher SFF scores than those reporting never having been diagnosed, but that those reporting a former diagnosis had lower SFF scores than those reporting a current diagnosis. Assuming that participants reporting former diagnoses are no more or less honest than those reporting a current diagnosis or no diagnosis, there are two possible explanations for this difference: 1) Those reporting a former diagnosis would have had SFF scores similar to participants with a current diagnosis when their own diagnosis had been current, which implies that feelings of fat are a marker of degree of recovery from an eating disorder; 2) Those who reported a former diagnosis had similar SFF scores to those they have now even when they had a current diagnosis, which implies that SFF scores are predictive of prognosis. Either possibility could be important from a clinical perspective but determining which accounts for the data will require further research.

The possibility of clinical applications of measures of feelings of fat raises the question of whether feelings of fat are only indicative of an eating disorder or whether they play a direct role in the development and maintenance of an eating disorder. It has been proposed that feelings of fat play a maintenance role in eating disorders (Fairburn [4]), so our results are consistent with that approach. Therefore, therapy targeting feelings of fat could be a useful part of treating eating disorders. Future research is necessary to address this question.

Our finding that feelings of fat are highly predictive of EDE-Q scores, and by extension eating disorders, may also have practical implications for screening for eating disorders. An intriguing implication of our results is that even the single item “I feel fat” may be a useful screen for potential eating disorders. This requires further exploration.

## Conclusions

In summary, our study developed a valid and coherent scale for measuring feelings of fat. Using this scale, we found that for three sample of university undergraduate women feelings of fat correlated extremely highly with evidence of disordered eating as measured by the EDE-Q. This finding has important clinical and theoretical implications, because it argues for feelings of fat having a central role in eating disorders. Our results suggest that further research into feelings of fat may yield useful insights into eating disorders.

## Data Availability

The data that support the findings of this study are available on request from the corresponding author.

## Abbreviations

ED: Eating Disorder
BMI: Body Mass Index
EDE-Q: Eating Disorders Examination – Questionnaire
SFF: State Feelings of Fat
